# Isolation and Screening of Indigenous Plant Growth-promoting Rhizobacteria from Different Rice Cultivars in Afghanistan Soils

**DOI:** 10.1264/jsme2.ME18168

**Published:** 2019-12-27

**Authors:** Safiullah Habibi, Salem Djedidi, Naoko Ohkama-Ohtsu, Wakil Ahmad Sarhadi, Katsuhiro Kojima, Roland V. Rallos, Maria Daniela Artigas Ramirez, Hiroko Yamaya, Hitoshi Sekimoto, Tadashi Yokoyama

**Affiliations:** 1 Faculty of Agriculture, Kabul University Afghanistan; 2 Institute of Agriculture, Tokyo University of Agriculture and Technology Japan; 3 Nuclear Research Institute 1101 Quezon Philippines; 4 Faculty of Agriculture, Utsunomiya University Japan

**Keywords:** rice, PGPR, IAA, ARA, phosphate and potassium solubilization, 16S rRNA

## Abstract

To develop biofertilizers for rice in Afghanistan, 98 plant growth-promoting rhizobacteria were isolated from rice plants and their morphological and physiological characteristics, such as indole-3-acetic acid production, acetylene reduction, phosphate and potassium solubilization, and siderophore production, were evaluated. The genetic diversity of these bacteria was also analyzed based on 16S rRNA gene sequences. Of 98 bacteria, 89.7% produced IAA, 54.0% exhibited nitrogenase activity, and 40% showed phosphate solubilization and siderophore production. Some isolates assigned to *Pseudomonas* (*brassicacearum*, *chengduensis*, *plecoglossicida*, *resinovorans*, and *straminea*) formed a relationship with rice, and *P. resinovorans* and *P. straminea* showed nitrogen fixation. *Rhizobium borbori* and *R. rosettiformans* showed a relationship with rice plants and nitrogen fixation. Among the isolates examined, AF134 and AF137 belonging to *Enterobacter ludwigii* and *P. putida* produced large amounts of IAA (92.3 μg mL^−1^) and exhibited high nitrogenase activity (647.4 nmol C_2_H_4_ h^−1^), respectively. In the plant growth test, more than 70% of the inoculated isolates showed significantly increased root and shoot dry weights. Highly diverse bacterial isolates showing promising rice growth-promoting traits were obtained from Afghanistan alkaline soils.

Rice is one of the most important staple foods for more than half of the world’s population (20), and accounts for 23% of the world’s calorie intake (6). Nitrogen (N) is one of the main limiting nutrients for crop productivity, including rice (27), and only one-third of the N applied as chemical fertilizer is used by rice plants (3). Phosphorus (P) and potassium (K) are major essential macronutrients for plants and are applied to soil in the form of phosphatic and potash fertilizers. However, a large portion of soluble inorganic P applied to soil as a chemical fertilizer is rapidly immobilized and becomes unavailable for plants (38).

Plant growth-promoting rhizobacteria (PGPR) were initially defined by Kloepper and Schroth (23) as a group of bacteria that colonize plant roots and exert beneficial effects on plant growth. They promote plant growth through direct and indirect mechanisms. Direct mechanisms are nitrogen fixation (4), the solubilization of nutrients, such as P (10) and K (21), siderophore production (25), phytohormone production (15, 39), and increasing crop tolerance to abiotic stress by producing chemical compounds, including enzymes (ACC–deaminase and chitinase), and substances, such as exopolysaccharides and rhizobitoxine (34). The indirect effects of PGPR on growth promotion occur against plant pathogens through several mechanisms, including induced systemic resistance (ISR), the production of antimicrobial compounds, and competition with pathogens for nutrients and colonization sites (11, 24).

PGPR have been extensively examined in an attempt to discover the most promising inoculants and develop biofertilizers and biocontrol agents (40) for various crops. Thus, many PGPR strains have been commercialized to promote plant growth (16). In many Asian countries, bio-fertilizer and bio-pesticide technologies are now in various stages of development and utilization (14, 36).

Rice is one of the most important staple crops in Afghanistan. It is widely grown in the North Eastern provinces. Large amounts of chemical fertilizers are required to obtain appropriate rice yields. Due to the environmental issues associated with the application of chemical fertilizers, the development of PGPR biofertilizers may be an effective and eco-friendly approach to reduce the use of chemical fertilizers and promote plant growth. Rice-associated PGPR have not yet been examined in Afghanistan soils, and, thus, the present study is the first to investigate rice-associated PGPR and develop biofertilizers for rice in Afghanistan.

## Materials and Methods

### Soil sampling

**Fig. 1:**
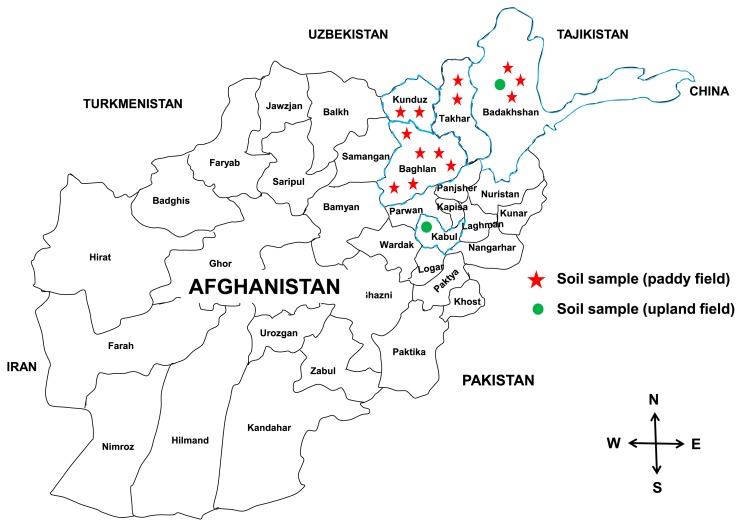
Map of Afghanistan showing soil sample collection sites.

Fifteen soil samples from different ecological zones and various fields (rice and legumes) in Afghanistan were collected at a depth of 0–20 cm (Fig. 1) and used to isolate PGPR. Thirteen soil samples belonged to paddy fields (rice rhizosphere) and two soil samples to upland fields (alfalfa and clover). These soil samples were used as inoculants to 5 rice cultivars.

### Isolation of PGPR

Regarding the isolation of PGPR, we followed the method described by Habibi *et al.* (19). The seeds of 5 rice cultivars (One Japanese [cv. Leaf star] and 4 Afghan [cv. Bala Doshi; cv. Monda Laghman; cv. Look Andarab and cv. Sorkhaq]) were surface-sterilized in 70% ethanol for 30 s, and in 3% sodium hypochlorite for 3 min. Twenty grams of soil from each soil sample was used as an inoculant to seeds in a pot containing sterilized vermiculite. Pots were kept in a growth chamber under controlled conditions (16-h light [250 μmol m^−2^ s ^−1^]/8-h dark photoperiod, at 25°C/18°C day/night temperatures). After 3 weeks, all plants were harvested, and 98 bacterial strains from the roots and leaves of rice plants were isolated using NFb semi-solid medium (12).

### Indole-3-acetic acid (IAA) production of isolates

In the IAA evaluation, NFb broth containing 100 mg L^−1^ L-tryptophan was inoculated by each strain, and incubated at 28°C for 2 d in the dark. Cell suspensions were then centrifuged at 9,730×*g* for 15 min to remove cells, and the concentration of IAA in the supernatant was measured using the Salkovski colorimetric technique (17) by measuring absorbance at 530 nm with a spectrophotometer (Ultrospec 3300 pro; Amersham Biosciences, Cambridge, United Kingdom). Cell density was assessed by the plate dilution method.

### Acetylene reduction assay (ARA) of isolates

In ARA, bacterial cultures were grown in vials containing N-free semisolid NFb medium and incubated at 28°C for 2 d. A total of 10% acetylene (v/v) was then injected into each vial and the cultures were further incubated at 30°C for 1 h. The concentration of ethylene in the vials was assessed using a Shimadzu 2014AF gas chromatograph (Shimadzu, Kyoto, Japan). Un-inoculated tubes were used as a negative control. The number of cells in each vial was measured by the plate dilution method.

### P- and K-solubilizing activities of isolates

Bacterial isolates were grown in NFb broth medium at 28°C for 48 h. Five microliters (10^6^ cells mL^−1^) of each culture was spotted onto Pikovskaia’s medium containing tricalcium phosphate (37) and slightly modified Aleksandrov medium (21). Plates were incubated at 28°C for 7 d. The P- and K-solubilizing activities (formation of a halozone or clear zone around bacterial colonies) of each isolate were evaluated by measuring the size of the halozone.

### Siderophore production by isolates

Bacterial isolates were grown in NFb broth medium at 28°C for 48 h. Five microliters (10^6^ cells mL^−1^) of each culture was spotted onto Chrome-azurol S (CAS) medium (2). Plates were incubated at 28°C for 2 d. The siderophore-solubilizing activity (formation of an orange or yellow halozone around colonies) of each isolate was evaluated by measuring the size of the orange or yellow zone around the colony.

### Molecular characterization

To assess genetic diversity, 81 (82.6% of the total) isolates were selected based on their physiological properties, host rice varieties, and climatic regions. These isolates were grown in NFb broth medium at 25°C for 4 d. Prior to genomic isolation, cells were harvested and washed twice with equal volumes of TNE buffer (10 mM Tris, 0.1 M NaCl, and 1 mM EDTA, pH 8). Genomic DNA was extracted from isolates using the method described by Yokoyama *et al.* (44). DNA concentrations and purities were examined using a NanoDrop 2000 UV-Vis spectrophotometer (Thermo Fisher Scientific, Wilmington, DE, USA).

### DNA amplification and sequencing

PCR amplification and sequencing of the 16S rRNA gene were conducted as described previously (19). The bacterial universal primers 27f (5′-AGTTTGATCCTGGCTC-3′) and 1525r (5′-AAG GAGGTGATCCAGCC-3′) were used to sequence the 16S rRNA gene. Amplifications were performed using 50-μL reaction mixtures containing 2 μM of the primer set 27f and 1525r, 0.5 μL Taq DNA Polymerase (ExTaq polymerase 5 U mL^−1^, Takara Bio, Otsu, Japan), 5 μL 10× reaction buffer, 4 μL dNTP mixture, and 1 μL DNA template (200–250 ng DNA). PCR products were checked by electrophoresis on a 1.5 (w/v) agarose gel. Amplified DNA bands corresponding to the 16S rRNA gene were purified using a QIAEX II agarose gel extraction kit (Qiagen, Valencia, CA, USA). Amplified DNA bands corresponding to the 16S rRNA gene were purified using a QIAEX II agarose gel extraction kit (Qiagen). Purified products were reacted using an ABI Prism BigDye Terminator v 3.1 cycle sequencing kit (Applied Biosystems, Foster City, CA, USA) and 27F and 1525r primers. Sequencing reaction mixtures were then analyzed using an ABI PRISM 3500 genetic analyzer (Applied Biosystems) according to the manufacturer’s protocols to obtain the DNA sequence of the 16S rRNA gene. Sequenced data were compared using the online software BLAST. Sequence alignment and construction of the phylogenetic tree were performed using MEGA version 6.06 (41).

### Effects of PGPR on rice growth

Based on physiological characteristics and sampling sites, 24 isolates were selected to evaluate their effects on rice growth. We used one Afghan rice cultivar (Bala Doshi) to evaluate the growth potential of selected isolates. Prior to sowing, 37 isolates were grown in 20 mL of NFb broth medium at 28°C for 2 d. The seeds of one Afghan rice cultivar (cv. Bala Doshi) were surface-sterilized as described in the PGPR isolation section. Germinated seeds were transplanted into pots containing gamma-irradiated (3 replicates) paddy field soil (185 g) (19), and each bacterial culture at a cell density of 10^9^ colony forming units mL^−1^ (CFU mL^−1^) was then applied to the seeds in the pot. All pots were transferred to a growth chamber controlled at 28°C±2°C during the day (16 h) and 25°C±2°C during the night (8 h). Each pot was irrigated with sterilized distilled water. Un-inoculated plants (negative control) and those inoculated with 2 strains: *Azosperillum brasilense* (Ts-13) (31) and *Bacillus pumilus* (TUAT-1), were used as positive controls. Plants were harvested after 3 weeks. The fresh weights of roots and shoots were recorded, and roots and shoots were then dried at 60°C for 2 d before dry weight measurements. The significance of differences between treatments and controls was analyzed using Tukey’s test (*P*<0.05).

## Results

### Isolation of PGPR

In the present study, the morphological characteristics of isolates were examined to evaluate colony diversity. The physiological characteristics of isolates were considered to differ more among the various colonies. Thus, based on the morphological characteristics of isolates, such as the form (circular, filamentous, and irregular), color (white, whitish, yellow, yellowish, creamy, and transplant), elevation (convex, flat, raised, crateriform, and umbonate), and margins (entire, filiform, and undulate) of colonies, 98 bacteria were selected and used in subsequent experiments. Soil samples and the number of isolates related to each province are shown in Table 1.

### Physiological properties of isolates

In the present study, some important physiological properties of PGPR, such as IAA production, nitrogen fixation, P solubilization, and K solubilization, were evaluated for some isolates (Table 2).

**Fig. 2:**
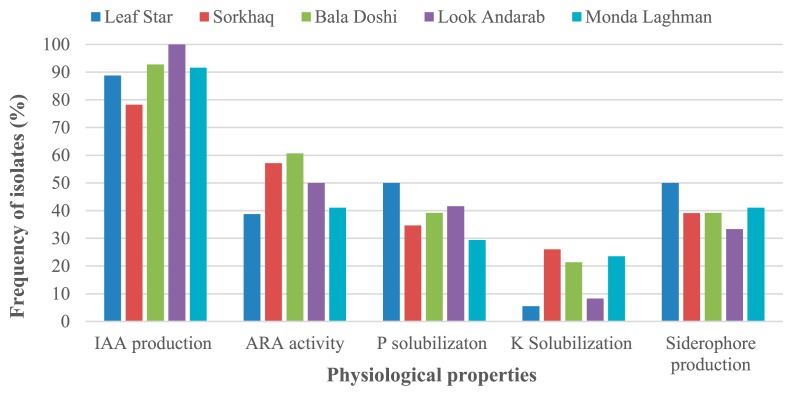
Frequencies of isolates from various rice varieties showing different physiological characteristics.

Among 98 isolates, 89.7% produced IAA, ranging between 2.0 and 92.3 μg mL^−1^. The frequencies of isolates related to each rice cultivar with the ability to produce IAA are shown in Fig. 2. Among all isolates, the AF134 isolate obtained from the leaves of Monda Laghman showed maximum IAA production (92.3 μg mL^−1^) followed by the AF135 (74.9 3 μg mL^−1^) isolate obtained from the roots of Sorkhaq (Table 2). Isolates obtained from Look-Andarab showed the highest frequency of IAA producers (100%) among the 5 rice varieties (Fig. 2).

Among 98 isolates, 54% exhibited ARA. Ethylene production rates varied widely among the isolates, and fluctuated between 0.1 and 647.4 nmol C_2_H_4_ h^−1^ 10^−6^ cells (Table 2). Bala Doshi isolates had a higher frequency of nitrogen-fixing bacteria (60.7%) than the 4 other rice cultivar isolates (Fig. 2). The AF137 isolate obtained from the leaves of Sorkhaq exhibited the highest nitrogenase activity (647.4 nmol C_2_H_4_ h ^−1^ 10^−6^) among all isolates (Table 2). Similarly, the AF84, AF51, AF30, AF75, and AF124 isolates exhibited higher ARA activity than the other isolates. These isolates were categorized as *R. daejeonense*.

The P solubilization abilities of 98 isolates were evaluated and we found that 39.8% of isolates exhibited the ability to show a clear zone around the colonies. The size of these clear zones fluctuated between 0.9 and 5.0 mm, and the AF43 isolate obtained from the roots of Bala Doshi displayed the largest clear zone (5.0 mm) among all isolates (Table 2). Regarding P solubilization activity, Leaf star isolates showed the highest frequency of P solubilizers (50%) among the 5 rice varieties (Fig. 2).

Among 98 isolates, 19.3% exhibited K solubilization activity. The size of the clear zones varied between 1.0 and 6.0 mm among the isolates. The AF13 isolate derived from the roots of Sorkhaq, showed the largest clear zone (6.0 mm) among all isolates (Table 2). Sorkhaq showed the highest frequency of effective K solubilizers (30.4%), whereas the lowest frequency of K solubilizers was found (5.5%) in Leaf Star among the rice varieties (Fig. 2).

Regarding siderophore production, 41 out of 98 isolates showed siderophore production via orange or yellow zone formation around the colonies. A high frequency of siderophore producers (50%) was observed in the Leaf Star rice variety (Fig. 2). AF95 and AF76 isolates from Leaf star and Bala Doshi showed a high potential for siderophore production (7.0 mm) (Table 2). AF5, 95, and 113 of Leaf star and AF46, 86, 96, and 100 of Bala Doshi showed higher siderophore production levels at more than 6.0 mm (Table 2).

### Genetic characterization

**Fig. 3:**
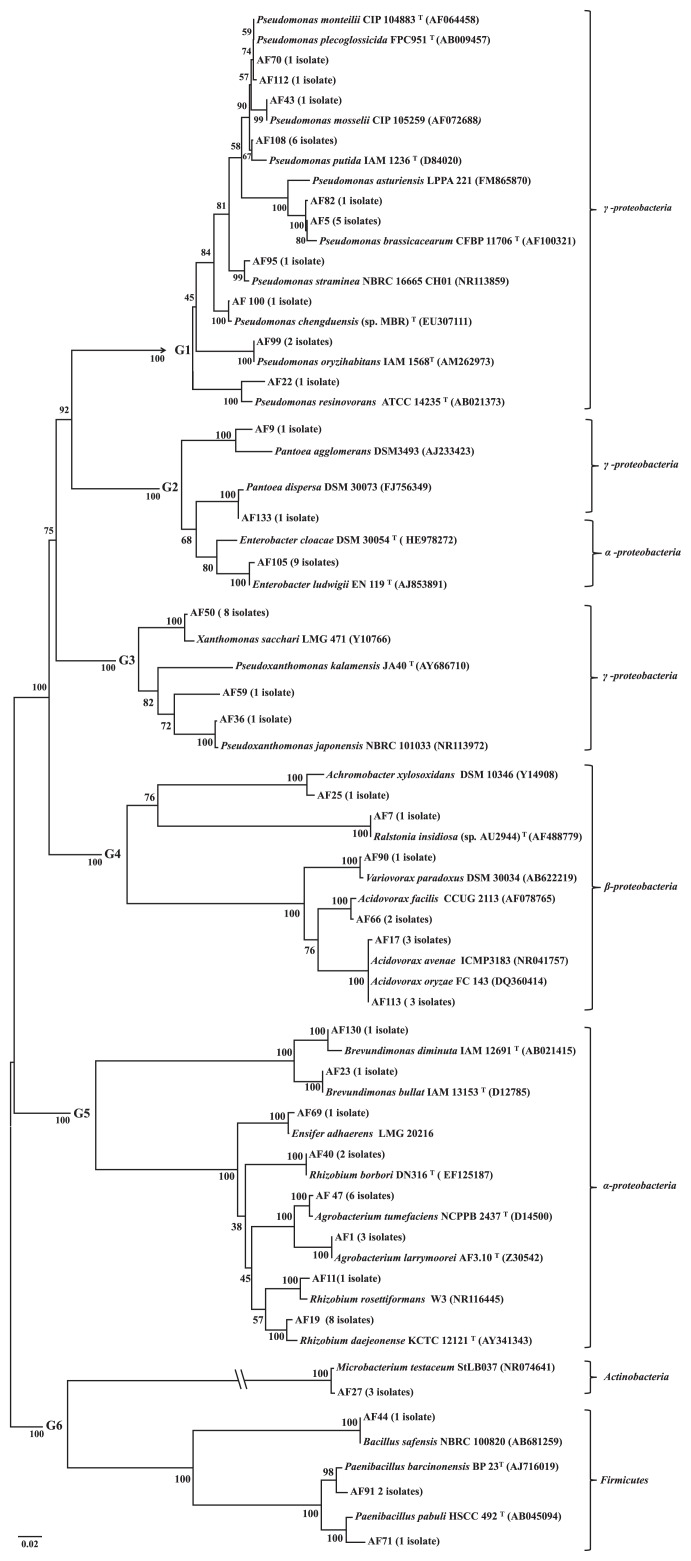
Phylogenetic trees of 16S rRNA constructed using partial nucleotide sequences (1,363 bp) from 81 isolates and type strains of species belonging to different genera. The numbers at the nodes indicate the level of bootstrap support, based on a neighbor-joining analysis.

Among 98 isolates, 81 were selected and a DNA fragment of approximately 1,363–1,456 bp from their 16S rRNA genes was sequenced and analyzed. Based on 16S rRNA sequences, 81 isolates were categorized into 16 different genera; *Acidovorax*, *Agrobacterium*, *Achromobacter*, *Bacillus*, *Brevundimonas*, *Ensifer*, *Enterobacter*, *Microbacterium*, *Paenibacillus*, *Pantoea*, *Pseudomonas*, *Pseudoxanthomonas*, *Ralstonia*, *Rhizobium*, *Variovorax*, and *Xanthomonas* (Fig. 3). The dominant genus was *Pseudomonas* (24.6%), followed by *Rhizobium* (13.5%). The 16S rRNA phylogenetic tree was divided into six groups (Fig. 3, G1–G6).

The G1 group included *P. putida*, *P. brassicacearum*, *P. oryzihabitans*, *P. chengduensis*, *P. monteilii*, *P. mosselii*, *P. plecoglossicida*, *P. resinovorans*, and *P. straminea*. Of the 6 isolates belonging to *P. putida*, the AF137 strain isolated from the leaves of the Sorkhaq rice cultivar showed the highest ARA activity among all isolates (Table 2). We detected 6 isolates of *P. brassicacearum*, including AF5, AF86, and AF46 (as shown in Table 2), in the roots and leaves of rice plants. To the best of our knowledge, this is the first study to show the relationship between an epiphyte and endophyte on rice plants. We also found AF100 (*P. chengduensis*), AF112 (*P. plecoglossicida*), AF22 (*P. resinovorans*), and AF95 (*P. straminea*) in the leaf part and AF43 (*P. mosselii*) in the root part of rice plants.

The G2 group consisted of two genera: *Pantoea* (2 isolates) and *Enterobacter* (9 isolates). These 11 isolates displayed high IAA production levels among all isolates. The G3 group comprised 10 isolates showing close similarities to *Xanthomonas* (8 isolates) and *Pseudoxanthomonas* (2 isolates) species. The G4 group contained 11 isolates related to different genera; *Achromobacter* (1 isolate), *Ralstonia* (1 isolate), *Variovorax* (1 isolate), and *Acidovorax* (8 isolates).

The G5 group of isolates included 4 genera (*Brevundimonas*, *Ensifer*, *Agrobacterium*, and *Rhizobium*). Among the 11 isolates related to the *Rhizobium* genus, 8 were categorized as *R. daejeonense*; two isolates (AF8 and AF40) as *R. borbori*, and one as *R. rosettiformans* (AF11). In the ARA evaluation, *R. daejeonense* isolates were found to be promising diazotrophs showing high ethylene production levels among all isolates (Table 2). Furthermore, the AF8 (*R. borbori*) and AF11 (*R. rosettiformans*) isolates exhibited nitrogenase activity under free living conditions (Table 2). The isolation of *R. borbori* from the leaves and *R. rosettiformans* from the roots of rice plants and their nitrogen fixation potentials is another novel result. We detected two *Brevundimonas* strains; one strain AF23 (*Brevundimonas bullata*) obtained from the leaves of Sorkhaq and another strain AF130 (*B. diminuta*) isolated from the roots of Bala Doshi. Both strains (AF23 and AF130) exhibited nitrogenase activity. To the best of our knowledge, the relationship between *B. bullata* and rice and the nitrogen fixation potentials of both strains (*B. diminuta* and *B. diminuta*) has been demonstrated here for the first time.

The G6 group of isolates showed high similarities to *Actinobacteria* (3 isolates) and *Firmicutes* ( *Bacillus* and *Paenibacillus*). We found three *Paenibacillus* strains; *P. pabuli* (AF71) isolated from Leaf Star in Badakhshan soils, and *P. bracinonensis* (AF91 and AF117) obtained from the roots of Sorkhaq in Baghlan soils. Both strains of *P. bracinonensis* (AF91 and AF117) exhibited nitrogenase activity.

### Effects of bacterial inoculation on rice growth

The inoculation effects of 24 isolates were evaluated on rice plants grown for 21 d. All bacterial isolates generally exerted positive effects on different plant growth parameters, as shown in Table 3. The isolate AF74 (*E. ludwigii*), which was isolated from the roots of Sorkhaq, significantly increased (*P*<0.05) shoot heights (55.7 cm) over those of the un-inoculated control. All of the isolates also exerted positive effects on root lengths. Root lengths varied between 7.3 and 15.6 cm, and significant variations were observed in response to the AF6 (*A. tumefaciens*), AF113 (*A. oryzae*), AF76 (*P. Putida*), AF46 (*P. brassicacearum*), and AF30 (*R. daejeonense*) isolates (Table 3). In shoot dry weight, 17 isolates (70.8% of the total) resulted in significant increases (*P*<0.05) in root dry weights over those of the un-inoculated plants. This enhancement varied between 60.55 and 260.3 mg plant^−1^, and the most significant increases were recorded for inoculated plants (221.1 to 260.3 mg plant^−1^) with AF74 (*E. ludwigii*), AF79 (*E. ludwigii*), AF46 (*P. brassicacearum*), AF42 (*E. ludwigii*), AF28 (*A. larrymoorei*), and AF112 (*P. plecoglossicida*) isolates (Table 3). Similarly, root dry weights exhibited significant increases in response to inoculations with 18 isolates (75.0%). The most significant increases were observed between 122.6 and 140.2 mg plant^−1^ following inoculations with the AF9 (*P. ananatis*), AF30 (*R. daejeonense*), AF52 (*A. larrymoorei*), AF74, and AF79 (*E. ludwigii*) isolates (Table 3). The isolates obtained from Bala Doshi rice cultivars promoted rice growth slightly more efficiently than the other rice cultivar isolates (Table 3).

## Discussion

In a previous study (19), we investigated the colonization of rice plants by different PGPR and compared their physiological characteristics and growth potentials related to each plant rhizosphere. In the present study, we examined the influence of various rice cultivars on PGPR colonization, and compared the physiological properties of these PGPR, which were obtained from different rice cultivars using different soil samples (mainly paddy fields).

IAA production is one of the important PGP traits of PGPR. The production of IAA by microbial isolates varies greatly among different species and strains of the same species and is also influenced by culture conditions, growth stages, and the availability of substrates (32). We assessed IAA production by 98 isolates obtained from different rice cultivars, and found that 100% of strains isolated from Look Andarab exhibited the ability to produce IAA among the 5 rice cultivars. Approximately 90% of the isolates from all rice cultivars (Leaf Star, Sorkhaq, Bala Doshi, Look Andarab, and Monda Laghman) showed the ability to produce IAA (Fig. 2). The reason why 100% of the isolates obtained from Look Andarab showed IAA production remains unclear; however, since Look Andarab is a cold-tolerant variety cultivated in temperate regions, this ability may be related to plant characteristics that allow for colonization by many IAA producers isolates. The largest amount of IAA produced was found in one endophytic PGPR, AF134 (*E. ludwigii*), which was isolated from the leaves of Monda Laghman (Table 2). Furthermore, the other isolates of *Enterobacter* species produced large amounts of IAA. Similarly, the potential of *Enterobacter* species to produce large amounts of IAA has been described in previous studies (19, 32, 39).

Nitrogen fixation by PGPR is another mechanism involved in plant growth promotion, and certain PGPR exhibit this potential. Associative diazotrophs are considered to play important roles in increasing plant productivity and decreasing the use of chemical fertilizers. In our assay, 60% of bacterial isolates from Bala Doshi exhibited nitrogenase activity; and this was the highest frequency of nitrogen-fixing bacteria among the five rice cultivars (Fig. 2). *R. daejeonense* isolates showed high nitrogen fixation activities among the studied isolates. Similarly, in a previous study, we isolated one effective nitrogen-fixing *R. daejeonense* from Japanese soil (19). *R. daejeonense* also appears to be a promising nitrogen-fixing bacteria in Afghan soils. AF137, an endophytic bacterium of rice leaves, showed the highest nitrogenase activity amongst all of the rice cultivar isolates examined (Table 2). Furthermore, AF7 isolated from the roots of Bala Doshi in Baghlan soils showed nitrogen fixation potential and IAA production. To the best of our knowledge, the isolation of *Ralstonia insidiosa* from rice plants and its ability to fix nitrogen has been demonstrated here for the first time.

P is the second important nutrient for plants and affects several characteristics of plant growth. P-solubilizing bacteria play an important role in releasing P from inorganic and organic pools in soil and provide P to plants via solubilization and mineralization. We assessed the P solubilization abilities of 98 isolates, and approximately 39% exhibited the ability to solubilize P mineral (tricalcium phosphate). AF43 (*P. mosselii*) obtained from the roots of Bala Doshi exhibited the highest P-solubilizing ability (Table 2). Similar findings on the P-solubilizing ability of *P. mosselii* have been reported (22, 35).

K is an essential plant nutrient that plants need for growth and reproduction. The majority of K in soil exists in various insoluble forms (rocks, minerals, and sedimentary materials), and K-solubilizing bacteria release solid K into available K, which is then directly absorbed by plants (45). However, under paddy field conditions, K solubilization may not be a major issue because PGPR may partially assist in the K-solubilizing process. In the present study, 19.4% of isolates exhibited K-solubilizing activity and the highest frequency of K solubilizers was observed in Sorkhaq isolates (30.4%). The AF13 isolate (*Enterobacter ludwigii*), which was obtained from the roots of Sorkhaq isolate rice cultivars, exhibited the maximum K-solubilizing activity among all isolates (Table 2). Furthermore, Zhang and Kong (45) recently reported the K-solubilizing activity of *Enterobacter* species (4 strains). The AF90 isolate (*Variovorax paradoxus*) obtained from the roots of Leaf star solubilized K mineral and produced siderophores (Table 2). To the best of our knowledge, the isolation of *V. paradoxus* from rice plants and the above mentioned physiological properties by that isolate have been described here for the first time.

Siderophores are low-molecular-weight iron chelators that directly promote plant growth by binding to ferric oxides and making them available for plants or indirectly by binding to the available forms of iron in soil and making them unavailable to pathogens (30). In our assay, 41.8% of isolates produced siderophores, and Leaf Star isolates showed the highest frequency of siderophore producers among the 5 rice cultivars (Fig. 2). *Pseudomonas* species were more active for siderophore production than the species of other genera (Table 2). The ability of *Pseudomonas* species to function as siderophore producers has been reported previously (1).

We sequenced the 16S rRNA genes of 81 isolates to assess PGPR diversity in Afghan soils (Supplementary material). The occurrence and distribution of microbial communities in the soil and rhizosphere are influenced by many factors, including root morphology, the stage of plant growth, root exudates, the physical and chemical properties of soil (5), plant species (18), soil type (9), soil depth (26), and cultivation practices (tillage/crop rotation) (29). An arid and semi-arid climate and Afghanistan topography resulted in different physical and chemical soil properties. Under these condition, the present results showed a higher distribution of *Pseudomonas* species in Afghan soils, particularly in paddy fields, than other bacterial genera. Regardless of the 17 non-characterized PGPR isolates in the present study, we found some clear differences among the colonization of rice cultivars by different genera of PGPR. For example, we did not detect *Xanthomonas* species in Bala Doshi or Leaf Star, *Agrobacterium* species in Sorkhaq, *Rhizobium* species in Monda Laghman, or *Acidovorax* species in Look Andarab. Among the 22 *Pseudomonas* species, 10 strains were found in Bala Doshi and four in Look Andarab (Supplementary material). The origin and common cultivation area of Bala Doshi and Look Andarab rice cultivars are related to the same province (Baghlan). Moreover, there were some PGPR unique to specific rice cultivars, such as *B. safensis* in Monda Laghman, *V. paradoxus* in Leaf Star, and *Ensifer adhaerens* in Bala Doshi isolates (Table 2). Many biotic and abiotic factors influence the colonization of plants by different or specific PGPR. Host specificity may be one factor influencing the colonization of PGPR to diverse crops. Regarding host specificity, Elbeltagy *et al.* (13) demonstrated that an endophytic bacterium (*Herbaspirillum* sp. strain B501) isolated from the stems of wild rice did not colonize cultivated rice after inoculation under aseptic conditions. Similarly, Bhattarai and Hess (7) reported that strains isolated from the same host plant were more efficient at improving plant growth.

Based on physiological characteristics and sampling sites, we selected 24 isolates for the plant growth test and evaluated their growth potential on rice plants. These isolates positively affected different growth parameters of rice plants. In the plant growth test, significantly greater increases in shoot and root dry weights were observed in *Pseudomonas* and *Rhizobium* species than in the species of other genera (Table 3). The potential of *Pseudomonas* and *Rhizobium* species as rice plant growth promoters has been reported in previous studies (8, 28, 33, 43). Furthermore, we found that PGPR isolated from Bala Doshi rice cultivars were significantly more effective at promoting plant growth than other rice cultivar isolates (Table 3). This may be due to the host specificity of Bala Doshi rice cultivars to the isolates obtained. As described above, Bhattarai and Hess reported similar findings (7).

In the present study, 98 bacterial strains were isolated from the leaves and roots of 5 rice cultivars using 15 soil samples as inoculants. We then examined the morphological, physiological, and genetic characteristics of these isolates to evaluate their potential as biofertilizers for rice crops. The results obtained revealed that AF74 (*E. ludwigii*), AF79 (*E. ludwigii*), AF46 (*P. brassicacearum*), AF112 (*P. plecoglossicida*), and AF30 (*Rhizobium daejeonense*) are potential candidates as biofertilizers for rice crops in Afghanistan. The use of biofertilizers may decrease the negative effects of chemical fertilizers on the environment and develop a sustainable agriculture in Afghanistan. However, further studies are required prior to the application of these PGPR in field conditions.

## SUPPLEMENTARY MATERIAL



## Figures and Tables

**Table 1:** Soil sample description and number of isolates obtained from each site of soil sampling.

Soil sampling sites	Number of soil samples	Latitude and longitude	Fields	pH^a^	Total number of isolates
Kabul	1	34° 31′ N–69° 11′ E	Clover	8.9±0.2	10
Baghlan	6	36° 08′ N–68° 42′ E	Paddy	8.0±0.1	36
Kunduz	2	36° 43′ N–68° 52′ E	Paddy	8.2±0.4	8
Takhar	2	35° 58′ N–70° 23′ E	Paddy	8.0±0.5	17
Badakhshan	4	36° 23′ N–71° 29′ E	Paddy and Alfalfa	8.2±0.1	27

a Measured with a pH meter in a 1:2.5 (w/v) soil and distilled water solution (42).

**Table 2:** Sampling sites, details of isolate origins, closest relatives, and physiological characteristics.

Rice varieties	Isolate name	Soil sampling site	Fields	Origin of isolates associated with rice	Closest relative based on 16S rRNA gene sequence¶	IAA production^a^	ARA^b^	c P-solubilization	d K-solubilization	e S-production	Accession numbers
Leaf star	AF5	Baghlan	Paddy	Leaf	*Pseudomonas brassicacearum* *(100%)	8.4±0.2	2.9±0.1	0.0	0.0	6.0±0.8	LC015569
	AF19	Baghlan	Paddy	Leaf	*Rhizobium daejeonense* (99%)	10.9±0.3	2.7±0.2	0.0	0.0	3.0±0.2	LC015583
	AF28	Baghlan	Paddy	Leaf	*Agrobacterium larrymoorei* (99%)	4.2±0.1	0.0	1.0±0.2	0.0	1.0±0.1	LC015600
	AF113	Baghlan	Paddy	Leaf	*Acidovorax oryzae* (100%)	2.8±0.4	0.0	3.0±0.1	0.0	6.0±0.8	LC015530
	AF52	Takhar	Paddy	Root	*Agrobacterium larrymoorei* (99%)	9.4±1.6	0.0	1.1±0.1	0.0	0.0	LC015601
	AF71	Badakhshan	Alfalfa	Root	*Paenibacillus pabuli* (99%)	4.2±0.7	0.0	0.0	0.0	5.0±0.7	LC015557
	AF90	Kunduz	Paddy	Root	*Variovorax paradoxus* (100%)	3.2±0.5	0.0	0.0	1.0±0.2	1.0±0.0	LC015538

Sorkhaq	AF9	Kunduz	Paddy	Leaf	*Pantoea ananatis* (100%)	22.5±1.2	3.4±0.3	2.5±0.1	4.0±0.5	1.0±0.2	LC015551
	AF11	Baghlan	Paddy	Leaf	*Rhizobium rosettiformans* (99%)	11.7±2.2	0.5±0.0	0.0	0.0	0.0	LC015582
	AF16	Kunduz	Paddy	Leaf	*Xanthomonas sacchari* (99%)	6.8±0.8	0.0	0.0	0.0	1.0±0.1	LC015607
	AF22	Badakhshan	Paddy	Leaf	*Pseudomonas resinovorans* (99%)	3.9±0.9	0.2±0.0	0.0	0.0	0.0	LC015561
	AF23	Takhar	Paddy	Leaf	*Brevundimonas bullata* (99%)	30.5±2.7	2.3±0.2	0.0	0.0	0.0	LC015540
	AF84	Baghlan	Paddy	Leaf	*Rhizobium daejeonense* (99%)	17.4±2.6	629.1±23.8	0.0	0.0	0.0	LC015590
	AF137	Kabul	Clover	Leaf	*Pseudomonas putida* (100%)	15.8±1.5	647.4±27.4	1.0±0.1	1.0±0.1	0.0	LC015575
	AF42	Badakhshan	Paddy	Root	*Enterobacter ludwigii* (99%)	17.1±0.4	3.9±0.1	2.0±0.2	2.0±0.1	1.0±0.1	LC015545
	AF74	Badakhshan	Paddy	Root	Enterobacter ludwigii (99%)	18.8±1.0	0.3±0.0	2.0±0.2	4.2±0.7	3.0±0.5	LC015546
	AF91	Baghlan	Paddy	Root	*Paenibacillus bracinonensis* (99%)	0.0	0.3±0.0	1.5±0.3	0.0	0.0	LC015555
	AF117	Baghlan	Paddy	Root	*Paenibacillus barcinonensis* (99%)	0.0	0.5±0.0	0.0	0.0	0.0	LC015556
	AF135	Kabul	Clover	Root	*Enterobacter ludwigii* (100%)	74.9±4.7	0.5±0.0	1.5±0.4	0.0	2.0±0.3	LC015542

Bala Doshi	AF100	Takhar	Paddy	Leaf	*Pseudomonas chengduensis* (99%)	0.0	0.0	0.0	0.0	6.0±0.7	LC015559
	AF51	Takhar	Paddy	Leaf	*Rhizobium daejeonense* (99%)	10.6±1.0	363.3±11.4	1.0±0.1	0.0	0.0	LC015586
	AF69	Takhar	Paddy	Leaf	*Ensifer adhaerens* (99%)	2.0±1.0	0.0	0.0	0.0	0.0	LC015581
	AF73	Takhar	Paddy	Leaf	*Pseudomonas putida* (100%)	7.4±0.8	14.1±1.1	1.5±0.4	0.0	6.5±0.4	LC015579
	AF86	Baghlan	Paddy	Leaf	*Pseudomonas brassicacearum* * (100%)10.0±2.4		0.8±0.0	0.0	0.0	6.0±0.9	LC015572
	AF6	Baghlan	Paddy	Leaf	*Agrobacterium tumefaciens* (99%)	16.5±0.9	0.0	1.0±0.3	0.0	0.0	LC015597
	AF79	Kunduz	Paddy	Leaf	*Enterobacter ludwigii* (99%)	47.4±3.0	0.4±0.1	1.0±0.1	0.0	1.0±0.1	LC015547
	AF30	Takhar	Paddy	Root	*Rhizobium daejeonense* (99%)	12.3±0.4	96.3±6.5	0.0	0.0	0.0	LC015585
	AF32	Kunduz	Paddy	Root	*Enterobacter ludwigii* (99%)	23.6±2.6	0.0	1.3±0.4	4.0±0.9	0.0	LC015544
	AF43	Takhar	Paddy	Root	*Pseudomonas mosselii* (100%)	7.6±0.8	0.0	5.0±0.6	0.0	3.0±0.5	LC015563
	AF46	Baghlan	Paddy	Root	Pseudomonas brassicacearum* (99%)	3.1±1.8	16.0±0.9	0.0	0.0	6.0±0.3	LC015571
	AF76	Takhar	Paddy	Root	*Pseudomonas putida* (100%)	9.9±1.4	1.0±0.1	0.0	0.0	7.0±0.9	LC015578
	AF7	Baghlan	Paddy	Root	*Ralstonia insidiosa* (100%)	2.7±0.4	2.8±0.2	0.0	0.0	0.0	LC015529
	AF8	Takhar	Paddy	Root	*Rhizobium borbori* (99%)	6.5±0.8	1.5±0.4	0.0	0.0	1.0±0.1	LC015592
	AF130	Baghlan	Paddy	Root	*Brevundimonas diminuta* (99%)	3.2±1.8	1.2±0.0	0.0	0.0	0.0	LC015539
	AF77	Baghlan	Paddy	Root	N.D.	2.1±0.6	0.2±0.0	0.9±0.2	0.0	0.0	N.D.
	AF96	Takhar	Paddy	Root	*Pseudomonas putida* (100%)	7.8±1.4	0.0	1.2±0.5	2.0±0.3	6.0±0.4	LC015580
	AF99	Kunduz	Paddy	Root	*Pseudomonas oryzihabitans* (99%)	6.5±0.6	1.0±0.0	0.0	0.0	4.0±0.9	LC015574

Look Andarab	AF54	Badakhshan	Paddy	Leaf	*Xanthomonas sacchari* (99%)	5.5±0.4	0.0	0.0	0.0	0.0	LC015609
	AF112	Badakhshan	Alfalfa	Leaf	*Pseudomonas plecoglossicida* (99%)	11.5±1.3	0.3±0.0	2.0±0.2	0.0	0.0	LC015560
	AF40	Takhar	Paddy	Root	*Rhizobium borbori* (99%)	10.8±0.9	1.7±0.2	0.0	0.0	0.0	LC015591
	AF75	Badakhshan	Paddy	Root	*Rhizobium daejeonense* (99%)	13.5±0.7	345.2±14.4	0.0	0.0	0.0	LC015589
	AF124	Badakhshan	Paddy	Root	*Rhizobium daejeonense* (99%)	19.2±3.4	218.3±23.5	0.0	0.0	0.0	LC015587

Monda Laghman	AF134	Kabul	Clover	Leaf	*Enterobacter ludwigii* (99%)	92.4±5.9	1.1±0.1	0.0	0.0	2.5±0.2	LC015549
	AF26	Takhar	Paddy	Root	*Agrobacterium tumefaciens* (100%)	8.6±0.5	0.0	0.0	1.0±0.2	0.0	LC015595
	AF105	Badakhshan	Paddy	Root	*Enterobacter ludwigii* (99%)	32.3±2.0	1.2±0.1	0.0	0.0	0.0	LC015541
	AF129	Badakhshan	Paddy	Root	Pseudomonas brassicacearum* (99%)	2.9±0.8	3.9±0.4	0.0	0.0	3.0±0.2	LC015570
	AF29	Badakhshan	Paddy	Root	*Xanthomonas sacchari* (99%)	8.4±2.5	0.0	0.0	4.0±0.8	4.0±0.3	LC015608
	AF26	Takhar	Paddy	Root	*Agrobacterium tumefaciens* (100%)	8.6±0.5	0.0	0.0	1.0±0.2	0.0	LC015595

a Amount of IAA produced (μg IAA per mL per 10^6^ cells).

b Acetylene reduction assay (ARA). Values represent activity expressed as nmol C_2_H_4_ h^−1^ 10^−6^ cells.

c P-solubilizing activity. Units represent size of clear zone (in mm) caused by dissolution of calcium phosphate.

d K-solubilizing activity. Units represent size of clear zone (in mm) caused by dissolution of potassium mineral.

e Siderophore production by bacterial strains. Units represent size of orange or yellow zone (in mm).

* *Pseudomonas brassicacearum* subsp. *Brassicacearum*.

¶ Values into parentheses indicate the percent of similarity between 16S RNA gene sequences of the isolates and those of known microorganisms of the NCBI GenBank.

N.D. means not determined.

**Table 3:** Summary effects of inoculating isolates on rice plants

Host rice varieties	Isolate names	Bacterial species¶	Origin of isolate associated with rice plant	Shoot height (cm)	Root length (cm)	Shoot dry weight (mg/plant)	Root dry weight (mg/plant)
	Control	.	.	43.6±2.1^a^	8.3±1.4	83.9±3.6	44.5±3.7

Leaf Star	AF19	*Rhizobium daejeonense* (99%)	Leaf	47.6±5.0	11.0±1.0	217.0±10.2*	95.5±9.3*
	AF28	*Agrobacterium larrymoorei* (99%)	Leaf	46.3±1.2	13.6±0.6	221.1±49.2*	98.2±12.1*
	AF113	*Acidovorax oryzae* (100%)	Leaf	50.3±2.5	15.7±2.1*	106.6±15.5	66.9±8.9
	AF52	*Agrobacterium larrymoorei* (99%)	Root	49.0±3.6	12.5±2.5	127.5±18.6	122.6±16.4*

Sorkhaq	AF9	*Pantoea ananatis* (100%)	Leaf	46.7±5.8	11.7±2.3	152.4±19.8	131.0±8.5*
	AF22	*Pseudomonas resinovorans* (99%)	Leaf	49.2±1.7	12.3±1.5	217.3±18.9*	114.8±18.5*
	AF16	*Xanthomonas sacchari* (99%)	Leaf	46.0±3.6	9.3±4.2	147.7±22.8	69.2±10.8
	AF42	*Enterobacter ludwigii* (99%)	Root	46.6±1.5	11.2±1.0	227.9±35.1*	107.7±11.3*
	AF74	*Enterobacter ludwigii* (99%)	Root	55.7±5.1*	12.7±0.6	260.3±12.3*	130.2±21.5*

Bala Doshi	AF20	*Rhizobium daejeonense* (99%)	Leaf	50.0±3.0	12.3±2.5	220.0±17.3*	95.9±9.7*
	AF6	*Agrobacterium tumefaciens* (99%)	Leaf	48.7±3.2	15.0±1.0*	166.6±10.6*	95.3±20.2*
	AF79	*Enterobacter ludwigii* (99%)	Leaf	48.3±2.1	10.6±1.2	249.8±19.8*	131.0±8.5*
	AF76	*Pseudomonas putida* (100%)	Root	51.6±1.5	13.3±2.5*	167.3±12.1*	73.7±7.4
	AF96	*Pseudomonas putida* (100%)	Root	46.7±1.5	13.3±0.6	211.6±12.0*	99.1±2.4*
	AF43	*Pseudomonas mosselii* (100%)	Root	49.0±2.6	12.3±0.6	161.3±16.9*	67.7±1.9
	AF46	*Pseudomonas brassicacearum* (99%)	Root	48.3±2.9	15.6±1.5*	239.4±9.2*	95.7±12.6*
	AF30	*Rhizobium daejeonense* (99%)	Root	48.6±3.2	14.7±2.3*	218.6±28.0*	140.2±13.9*
	AF99	*Pseudomonas oryzihabitans* (99%)	Root	48.6±3.1	12.3±0.6	195.1±24.6*	102.3±18.7*

Look Andarab	AF54	*Xanthomonas sacchari* (99%)	Leaf	48.0±1.0	13.3±2.9	203.9±16.9*	94.1±8.8*
	AF114	*Agrobacterium tumefaciens* (100%)	Leaf	42.0±4.0	14.6±1.2	118.9±11.6	74.7±11.0
	AF112	*Pseudomonas plecoglossicida* (99%)	Leaf	50.6±3.8	14.3±2.3	226.0±22.8*	104.3±18.9*
	AF124	*Rhizobium daejeonense* (99%)	Root	50.7±0.6	13.5±1.3	205.1±24.0*	103.2±9.4*

Monda Laghman	AF105	*Enterobacter ludwigii* (99%)	Root	43.7±5.8	8.7±2.1	93.7±12.3	49.0±1.8
	AF29	*Xanthomonas sacchari* (99%)	Root	47.3±9.9	11.0±2.6	155.1±25.5	90.0±4.8*

Positive controls	TUAT1	*Bacillus pumilus*	.	51.3±4.5	11.7±1.4	253.2±16.7*	107.3±10.6*
	TS-13	*Azospirillum brasilense*	.	50.3±3.5	11.3±0.6	223.3±20.8*	103.3±5.8*

a Mean value (*n*=3 replicates)

* Value is significantly different from the control, within each column (*P*<0.05)

¶ Values into parentheses indicate the percent of similarity between 16S RNA gene sequences of the isolates and those of known microorganisms of the NCBI GenBank.

## Abbreviations

ARA: Acetylene reduction assay
BLAST: Basic local alignment search tool
CTAB: Hexadecyltrimethylammonium bromide
DDBJ: DNA data bank Japan
DNA: Deoxyribonucleic acid
FFTC: Food and fertilizer technology center
IAA: Indole-3-acetic acid
MEGA: Molecular Evolutionary Genetics Analysis
PCR: Polymerase chain reaction
PGP: Plant growth-promoting
PGPR: Plant growth-promoting rhizobacteria
