# Supplementary material for: Isolation and Screening of Indigenous Plant Growth-promoting Rhizobacteria from Different Rice Cultivars in Afghanistan Soils
Source: Microbes Environ. 2019 Dec 27;34(4):347–55. doi: 10.1264/jsme2.ME18168 (PMC6934389; doi:10.1264/jsme2.ME18168)
Supplement: Supplementary file 1 [file 34_347_s1.pdf]

Table 2 (Supplementary)

Rice varieties	Isolate name	Soil sampling site	Fields	Origin of isolates associated with	Closest relative based on 16S rRNA gene [¶]	IAA production [a]	ARA [b]	[c] P-solubilization	[d] K-solubilization	[e] S-production	Accession Numbers
Leaf star	AF1	Baghlan	Paddy	Leaf	*Agrobacterium larrymoorei* (99%)	14.5±0.7	0.0	1.0±0.1	0.0	0.0	LC015599
	AF5	Baghlan	Paddy	Leaf	*Pseudomonas brassicacearum* * (100%)	8.4±0.2	2.9±0.1	0.0	0.0	6.0±0.8	LC015569
	AF19	Baghlan	Paddy	Leaf	*Rhizobium daejeonense* (99%)	10.9±0.3	2.7±0.2	0.0	0.0	3.0±0.2	LC015583
	AF21	Badakhshan	Alfalfa	Leaf	N.D.	0.0	1.3±0.0	0.0	0.0	0.0	N.D.
	AF27	Takhar	Paddy	Leaf	*Microbacterium testaceum* (99%)	7.3±0.1	0.0	1.0±0.1	0.0	0.0	LC015552
	AF28	Baghlan	Paddy	Leaf	*Agrobacterium larrymoorei* (99%)	4.2±0.1	0.0	1.0±0.2	0.0	1.0±0.1	LC015600
	AF57	Baghlan	Paddy	Leaf	N.D.	5.6±0.3	0.2±0.1	0.0	0.0	0.0	N.D.
	AF80	Kabul	Clover	Leaf	*Microbacterium testaceum* (99%)	5.9±0.4	0.0	1.5±0.4	0.0	0.0	LC015554
	AF95	Takhar	Paddy	Leaf	*Pseudomonas straminea* (100%)	5.3±0.2	5.0±0.2	0.0	0.0	7.0±0.5	LC015565
	AF113	Baghlan	Paddy	Leaf	*Acidovorax oryzae* (100%)	2.8±0.4	0.0	3.0±0.1	0.0	6.0±0.8	LC015530
	AF52	Takhar	Paddy	Root	*Agrobacterium larrymoorei* (99%)	9.4±1.6	0.0	1.1±0.1	0.0	0.0	LC015601
	AF62	Badakhshan	Alfalfa	Root	N.D.	9.5±1.0	0.3±0.0	1.2±0.3	0.0	0.0	N.D.
	AF70	Takhar	Paddy	Root	*Pseudomonas monteilii* (100%)	17.7±0.4	0.0	0.0	0.0	5.0±0.9	LC015566
	AF71	Badakhshan	Alfalfa	Root	*Paenibacillus pabuli* (99 %)	4.2±0.7	0.0	0.0	0.0	5.0±0.7	LC015557
	AF78	Baghlan	Paddy	Root	*Pseudomonas brassicacearum* * (99%)	10.5±2.1	0.0	0.0	0.0	0.0	LC015568
	AF87	Badakhshan	Paddy	Root	*Rhizobium daejeonense* (99%)	14.8±0.5	0.1±0.0	1.1±0.3	0.0	0.0	LC015588
	AF90	Kunduz	Paddy	Root	*Variovorax paradoxus* (100%)	3.2±0.5	0.0	0.0	1.0 ± 0.2	1.0 ± 0.0	LC015538
	AF17	Baghlan	Paddy	Root	*Acidovorax avenae* (100%)	0.0	0.0	1.5±0.3	0	4.0±0.5	LC015533
Sorkhaq	AF9	Kunduz	Paddy	Leaf	*Pantoea ananatis* (100%)	22.5±1.2	3.4±0.3	2.5±0.1	4.0 ± 0.5	1.0±0.2	LC015551
	AF10	Baghlan	Paddy	Leaf	*Xanthomonas sacchari* (100%)	8.0±0.9	0.0	0.0	0.0	0.0	LC015604
	AF11	Baghlan	Paddy	Leaf	*Rhizobium rosettiformans* (99%)	11.7±2.2	0.5±0.0	0.0	0.0	0.0	LC015582
	AF16	Kunduz	Paddy	Leaf	*Xanthomonas sacchari* (99%)	6.8±0.8	0.0	0.0	0.0	1.0±0.1	LC015607
	AF22	Badakhshan	Paddy	Leaf	*Pseudomonas resinovorans* (99%)	3.9±0.9	0.2±0.0	0.0	0.0	0.0	LC015561
	AF23	Takhar	Paddy	Leaf	*Brevundimonas bullata* (99%)	30.5±2.7	2.3±0.2	0.0	0.0	0.0	LC015540
	AF33	Baghlan	Paddy	Leaf	N.D.	9.2±3.1	1.3±0.2	0.0	0.0	2.0±0.4	N.D.
	AF39	Kabul	Clover	Leaf	*Acidovorax oryza* (100%)	3.2±1.1	3.2±0.2	0.0	0.0	3.0±0.4	LC015531
	AF84	Baghlan	Paddy	Leaf	*Rhizobium daejeonense* (99%)	17.4±2.6	629.1±23.8	0.0	0.0	0.0	LC015590
	AF118	Badakhshan	Alfalfa	Leaf	N.D.	0.0	0.0	1.3±0.1	0.0	0.0	N.D.
	AF136	Kabul	Clover	Leaf	N.D.	5.0±0.8	4.4±0.1	0.0	0.0	0.0	N.D.

	AF137	Kabul	Clover	Leaf	*Pseudomonas putida* (100%)	15.8±1.5	647.4±27.4	1.0±0.1	1.0±0.1	0.0	LC015575
	AF3	Baghlan	Paddy	Root	N.D.	0.0	0.0	0.0	0.0	0.0	N.D.
	AF13	Baghlan	Paddy	Root	*Enterobacter ludwigii* (99%)	36.9±2.1	0.1±0.0	1.0±0.2	6.0±0.5	1.0±0.1	LC015543
	AF15	Kunduz	Paddy	Root	*Xanthomonas sacchari* (99%)	7.4±0.9	0.0	0.0	0.0	2.0±0.3	LC015606
	AF42	Badakhshan	Paddy	Root	*Enterobacter ludwigii* (99%)	17.1±0.4	3.9±0.1	2.0±0.2	2.0±0.1	1.0±0.1	LC015545
	AF50	Badakhshan	Paddy	Root	*Xanthomonas sacchari* (99%)	7.5±2.2	0.0	0.0	0.0	2.0±0.2	LC015602
	AF91	Baghlan	Paddy	Root	*Paenibacillus bracinonensis* (99%)	0.0	0.3±0.0	1.5±0.3	0.0	0.0	LC015555
	AF115	Baghlan	Paddy	Root	N.D.	0.0	0.0	0.0	2.0±0.3	0.0	N.D.
	AF117	Baghlan	Paddy	Root	*Paenibacillus barcinonensis* (99%)	0.0	0.5±0.0	0.0	0.0	0.0	LC015556
	AF74	Badakhshan	Paddy	Root	*Enterobacter ludwigii* (99%)	18.8±1.0	0.3±0.0	2.0±0.2	4.2±0.7	3.0±0.5	LC015546
	AF127	Baghlan	Paddy	Root	N.D.	11.4±1.6	1.8±0.0	1.0±0.1	5.0±0.7	0.0	N.D.
	AF135	Kabul	Clover	Root	*Enterobacter ludwigii* (100%)	74.9±4.7	0.5±0.0	1.5±0.4	0.0	2.0±0.3	LC015542
Bala Doshi	AF6	Baghlan	Paddy	Leaf	*Agrobacterium tumefaciens* (99%)	16.5±0.9	0.0	1.0±0.3	0.0	0.0	LC015597
	AF20	Baghlan	Paddy	Leaf	*Rhizobium daejeonense* (99%)	10.0±1.4	16.7±2.1	1.2±0.2	0.0	0.0	LC015584
	AF24	Takhar	Paddy	Leaf	N.D.	8.8±0.3	0.0	0.0	0.0	0.0	N.D.
	AF25	Takhar	Paddy	Leaf	*Anchrobacter xylosoxidans* (99%)	6.6±0.5	2.8±0.1	0.0	0.0	0.0	LC015528
	AF100	Takhar	Paddy	Leaf	*Pseudomonas chengduensis* (99%)	0.0	0.0	0.0	0.0	6.0±0.7	LC015559
	AF51	Takhar	Paddy	Leaf	*Rhizobium daejeonense* (99%)	10.6±1.0	363.3±11.4	1.0±0.1	0.0	0.0	LC015586
	AF69	Takhar	Paddy	Leaf	*Ensifer adhaerens* (99%)	2.0±1.0	0.0	0.0	0.0	0.0	LC015581
	AF73	Takhar	Paddy	Leaf	*Pseudomonas putida* (100%)	7.4±0.8	14.1±1.1	1.5±0.4	0.0	6.5±0.4	LC015579
	AF79	Kunduz	Paddy	Leaf	*Enterobacter ludwigii* (99%)	47.4±3.0	0.4±0.1	1.0±0.1	0.0	1.0±0.1	LC015547
	AF83	Baghlan	Paddy	Leaf	N.D.	13.9±0.6	0.6±0.0	0.0	3.0±0.8	1.0±0.3	N.D.
	AF86	Baghlan	Paddy	Leaf	*Pseudomonas brassicacearum* * (100%)	10.0±2.4	0.8±0.0	0.0	0.0	6.0±0.9	LC015572
	AF7	Baghlan	Paddy	Root	*Ralstonia insidiosa* (100%)	2.7±0.4	2.8±0.2	0.0	0.0	0.0	LC015529
	AF8	Takhar	Paddy	Root	*Rhizobium borbori* (99%)	6.5±0.8	1.5±0.4	0.0	0.0	1.0±0.1	LC015592
	AF30	Takhar	Paddy	Root	*Rhizobium daejeonense* (99%)	12.3±0.4	96.3±6.5	0.0	0.0	0.0	LC015585
	AF31	Baghlan	Paddy	Root	*Pseudomonas oryzihabitans* (99%)	36.9±1.2	0.71±0.1	0.0	0.0	0.0	LC015573
	AF32	Kunduz	Paddy	Root	*Enterobacter ludwigii* (99%)	23.6±2.6	0.0	1.3±0.4	4.0±0.9	0.0	LC015544
	AF43	Takhar	Paddy	Root	*Pseudomonas mosselii* (100%)	7.6±0.8	0.0	5.0±0.6	0.0	3.0±0.5	LC015563
	AF46	Baghlan	Paddy	Root	*Pseudomonas brassicacearum* * (99%)	3.1±1.8	16.0±0.9	0.0	0.0	6.0±0.3	LC015571
	AF55	Takhar	Paddy	Root	*Acidovorax facilis* (99%)	4.1±1.5	0.3±0.0	0.0	0.0	0.0	LC015537
	AF66	Badakhshan	Paddy	Root	*Acidovorax facilis* (99%)	2.4±0.5	0.0	0.0	0.0	0.0	LC015536
	AF76	Takhar	Paddy	Root	*Pseudomonas putida* (100%)	9.9±1.4	1.0±0.1	0.0	0.0	7.0±0.9	LC015578
	AF77	Baghlan	Paddy	Root	N.D.	2.1±0.6	0.2±0.0	0.9±0.2	0.0	0.0	N.D.

	AF96	Takhar	Paddy	Root	*Pseudomonas putida* (100%)	7.8±1.4	0.0	1.2±0.5	2.0±0.3	6.0±0.4	LC015580
	AF97	Badakhshan	Paddy	Root	N.D.	2.9±0.7	0.0	0.0	3.0±0.5	0.0	N.D.
	AF98	Baghlan	Paddy	Root	N.D.	5.7±0.3	0.0	1.5±0.3	4.0±1.0	0.0	N.D.
	AF99	Kunduz	Paddy	Root	*Pseudomonas oryzihabitans* (99%)	6.5±0.6	1.0±0.0	0.0	0	4.0±0.9	LC015574
	AF108	Baghlan	Paddy	Root	*Pseudomonas putida* (100%)	0.0	0.0	1.5±0.2	2.0±0.2	0.0	LC015577
	AF130	Baghlan	Paddy	Root	*Brevundimonas diminuta* (99%)	3.2±1.8	1.2 ± 0.0	0.0	0.0	0.0	LC015539
Look Andarab	AF36	Badakhshan	Paddy	Leaf	*Pseudoxanthomonas mexicana* (100%)	2.4±0.2	16.9±0.9	1.1±0.1	0.0	0.0	LC015562
	AF54	Badakhshan	Paddy	Leaf	*Xanthomonas sacchari* (99%)	5.5±0.4	0.0	0.0	0.0	0.0	LC015609
	AF60	Badakhshan	Paddy	Leaf	*Microbacterium testaceum* (99%)	3.2±0.2	0.0	1.0±0.1	0.0	0.0	LC015553
	AF112	Badakhshan	Alfalfa	Leaf	*Pseudomonas plecoglossicida* (99%)	11.5±1.3	0.3±0.0	2.0±0.2	0.0	0.0	LC015560
	AF114	Badakhshan	Alfalfa	Leaf	*Agrobacterium tumefaciens* (100%)	14.1±0.9	0.0	0.0	0.0	0.0	LC015594
	AF38	Baghlan	Paddy	Root	N.D.	4.9±0.7	0.0	0.0	0.0	2.0±0.3	N.D.
	AF40	Takhar	Paddy	Root	*Rhizobium borbori* (99%)	10.8±0.9	1.7±0.2	0.0	0.0	0.0	LC015591
	AF75	Badakhshan	Paddy	Root	*Rhizobium daejeonense* (99%)	13.5±0.7	345.2±14.4	0.0	0.0	0.0	LC015589
	AF82	Baghlan	Paddy	Root	*Pseudomonas brassicacearum* * (99%)	7.2±0.8	0.9±0.0	0.0	0.0	5.0±0.6	LC015567
	AF106	Baghlan	Paddy	Root	*Pseudomonas putida* (100%)	12.8±1.8	0.0	1.0±0.1	3.0±0.5	5.0±0.8	LC015576
	AF124	Badakhshan	Paddy	Root	*Rhizobium daejeonense* (99%)	19.2±3.4	218.3±23.5	0.0	0.0	0.0	LC015587
	AF119	Badakhshan	Paddy	Root	*Xanthomonas sacchari* (99%)	6.7±0.4	0.0	1.0±0.1	0.0	2.0±0.3	LC015603
Monda Laghman	AF132	Kabul	Clover	Leaf	*Acidovorax avenae* (100%)	9.9±1.1	0.0	1.0±0.3	3.0±0.7	1.0 ± 0.1	LC015534
	AF134	Kabul	Clover	Leaf	*Enterobacter ludwigii* (99%)	92.4±5.9	1.1±0.1	0.0	0.0	2.5±0.2	LC015549
	AF138	Kabul	Clover	Leaf	*Acidovorax avenae* (100%)	4.0±1.2	0.0	0.0	0.0	0.0	LC015535
	AF26	Takhar	Paddy	Root	*Agrobacterium tumefaciens* (100%)	8.6±0.5	0.0	0.0	1.0±0.2	0.0	LC015595
	AF29	Badakhshan	Paddy	Root	*Xanthomonas sacchari* (99%)	8.4±2.5	0.0	0.0	4.0±0.8	4.0±0.3	LC015608
	AF44	Baghlan	Paddy	Root	*Bacillus safensis* (100%)	3.8±0.3	1.7±0.2	2.0±0.2	0.0	0.0	LC015558
	AF48	Badakhshan	Paddy	Root	*Acidovorax oryzae* (100%)	6.4±0.7	0.0	0.0	0.0	0.0	LC015532
	AF49	Badakhshan	Alfalfa	Root	N.D.	9.4±1.0	1.1±0.3	0.0	0.0	0.0	N.D.
	AF58	Badakhshan	Alfalfa	Root	*Agrobacterium tumefaciens* (100%)	15.9±1.3	0.0	1.0±0.1	0.0	0.0	LC015598
	AF59	Badakhshan	Alfalfa	Root	*Pseudoxanthomonas japonensis* (99%)	7.8±0.5	0.0	0.0	0.0	5.0±0.4	LC015564
	AF88	Badakhshan	Paddy	Root	N.D.	0.0	0.0	1.1±0.1	0.0	0.0	N.D.
	AF105	Badakhshan	Paddy	Root	*Enterobacter ludwigii* (99%)	32.3±2.0	1.2±0.1	0.0	0.0	0.0	LC015541
	AF116	Badakhshan	Paddy	Root	*Xanthomonas sacchari* (100%)	9.5±2.0	0.0	0.0	0.0	3.0±0.5	LC015605
	AF121	Badakhshan	Alfalfa	Root	*Agrobacterium tumefaciens* (100%)	13.9±1.3	0.0	0.0	0.0	0.0	LC015596
	AF129	Badakhshan	Paddy	Root	*Pseudomonas brassicacearum* (99%)	2.9±0.8	3.9±0.4	0.0	0.0	3.0±0.2	LC015570
	AF133	Kabul	Clover	Root	*Pantoea dispersa* (99%)	35.5±2.5	1.6 ± 0.0	0.0	0.0	1.0±0.2	LC015550

AF139	Kabul	Clover	Root	*Enterobacter ludwigii* (99%)	46.8±4.0	0.4±0.0	1.2±0.3	1.0±0.1	0.0	LC015548

[a] Amount of IAA produced (µg IAA per mL per 10^6 cells).

[b] Acetylene reduction assay (ARA). Values represent activity expressed as nmol C_2H_4 / h/ 10^6 cells.

[c] Phosphate-solubilizing activity. Units represent size of clear zone (in mm) caused by dissolution of calcium phosphate.

[d] Potassium-solubilizing activity. Units represent size of clear zone (in mm) caused by dissolution of potassium mineral.

[e] Siderophore production by bacterial strains. Units represent size of orange or yellow zone (in mm).

[*] *Pseudomonas brassicacearum* subsp. *brassicacearum*

[¶] Values into parentheses indicate the percent of similarity between 16S RNA gene sequences of the isolates and those of known microorganisms of the NCBI GenBank.

N.D. means not determined
